# BOTRYTIS-INDUCED KINASE1, a plasma membrane-localized receptor-like protein kinase, is a negative regulator of phosphate homeostasis in *Arabidopsis thaliana*

**DOI:** 10.1186/s12870-016-0841-1

**Published:** 2016-07-07

**Authors:** Huijuan Zhang, Lei Huang, Yongbo Hong, Fengming Song

**Affiliations:** College of Life Science, Taizhou University, Taizhou, Zhejiang 318001 People’s Republic of China; National Key Laboratory for Rice Biology, Institute of Biotechnology, Zhejiang University, Hangzhou, 310058 People’s Republic of China

**Keywords:** BOTRYTIS-INDUCED KINASE1 (BIK1), Phosphate starvation stress, Phosphate starvation response, Root architecture

## Abstract

**Background:**

Plants have evolved complex coordinated regulatory networks to cope with deficiency of phosphate (Pi) in their growth environment; however, the detailed molecular mechanisms that regulate Pi sensing and signaling pathways are not fully understood yet. We report here that the involvement of Arabidopsis BIK1, a plasma membrane-localized receptor-like protein kinase that plays critical role in immunity, in Pi starvation response.

**Results:**

qRT-PCR analysis revealed that expression of *BIK1* was induced by Pi starvation and GUS staining indicated that the *BIK1* promoter activity was detected in root, stem and leaf tissues of plants grown in Pi starvation condition, demonstrating that *BIK1* is responsive to Pi starvation stress. The *bik1* plants accumulated higher Pi content in root and leaf tissues and exhibited altered root architecture such as shorter primary roots, longer and more root hairs and lateral roots, as compared with those in the wild type plants, when grown under Pi sufficient and deficient conditions. Increased anthocyanin content and acid phosphatase activity, reduced accumulation of reactive oxygen species and downregulated expression of Pi starvation-induced genes including *PHR1*, *WRKY75*, *AT4*, *PHT1;2* and *PHT1;4* were observed in *bik1* plants grown under Pi deficient condition. Furthermore, the expression of *PHO2* was downregulated while the expression of *miRNA399a* and *miRNA399d*, which target to *PHO2*, was upregulated in *bik1* plants, compared to the wild type plants, when grown under Pi deficient condition.

**Conclusion:**

Our results demonstrate that *BIK1* is a Pi starvation-responsive gene that functions as a negative regulator of Pi homeostasis in Arabidopsis.

## Background

Phosphate (Pi) is one of the indispensable macronutrients to plants for growth, development and reproduction. Pi deficiency (−Pi) is one of the main limiting factors for increasing crop yield and improving quality because of low bioavailability of Pi in soil [1–4]. To cope with Pi deficiency, plants develop a series of tightly controlled adaptive responses including external developmental alterations of increasing Pi absorption and internal metabolic, physiological and biochemical alterations of reducing Pi usage [4–6]. To increase Pi uptake under Pi depletion stress, plants adapt to modulate the root architecture bearing more and longer lateral roots as well as denser root hairs, which enable the roots to explore the Pi resources in soil [7–12]. To reduce Pi consumption under Pi deficiency condition, plants often modulate metabolisms to maintain intracellular Pi homeostasis by reducing metabolic consumption of Pi [1, 3, 13–15] and degrading compounds to release Pi [16]. Furthermore, a series of physiological and biochemical adaptions including the induction and secretion of phosphatases and organic acids and accumulation of protective metabolites such as anthocyanin help augment the availability of both endogenous and exogenous Pi [1, 4, 17, 18].

Genetic, physiological and biochemical studies in Arabidopsis have demonstrated that the acquisition, allocation, and metabolism of Pi are highly regulated processes and require the concerted action of multiple membrane Pi transport systems [19–23]. Among five distinct classes of proteins possessing Pi transport activity, the plastidic Pi translocator group function as antiport systems, whereas the other four Pi transporter families, named the PHOSPHATE TRANSPORTER1 (Pht1), Pht2, Pht3 and Pht4, contribute to the acquisition, allocation and remobilization of Pi [24–27]. Most of the plasma membrane-localized high affinity transporters in the Pht1 family mediate Pi acquisition from external environment [1, 24, 28–30]. Once Pi is transported into root epidemic cells, translocation and allocation of Pi within the plants and cells are key steps in maintaining Pi homeostasis at cell and whole plant levels. The low affinity transporters in the Pht2 and Pht4 families are thought to participate mainly in Pi transfer across internal cellular membranes and thus allocate Pi in different compartments of the cells [25, 27, 31–33]. Pht1;5, a Pht1 family member, PHO1, Pht1;8 and Pht1;9 were shown to play critical roles in systemic regulation of Pi homeostasis, e.g. mobilization of Pi from source to sink organs in accordance with the Pi status of the plant [34–36].

Functional characterization of genes in a number of mutants with altered response to Pi depletion have led to the identification of several different regulatory mechanisms controlling the adaptive responses to Pi starvation. These regulatory mechanisms include transcriptional regulation by transcription factors such as PHR1, WRKY6, WRKY42, WRKY45, WRKY75, ZAT6, bHLH32, ERF070 and MYB62 [37–47], posttranscriptional regulation by microRNAs including miRNA399 [48–53], posttranslational regulation by protein modifications such as sumoylation of SIZ1 [54], phosphorylation of Pht1.1 and activation of MKK9-MPK3/MPK6 module [55, 56], deubiquitination of UBP14 [57] and chromatin histone modification and epigenetic [46, 58]. Furthermore, it was also demonstrated that Pi starvation response cross-talks signaling mediated by different hormones such as auxin [10, 15, 59–61], cytokinin [10, 15, 62, 63], ethylene [9, 10, 64–66] and gibberellin [67]. Although great advance on the adaptive response to Pi starvation has been made during the last decade, the molecular mechanism that regulates these adaptive processes has yet to be elucidated in detail.

The *Arabidopsis thaliana BOTRYTIS-INDUCED KINASE1* (*BIK1*) encodes a plasma membrane-localized receptor-like protein kinase and plays critical roles in *Botrytis cinerea* resistance [68]. Recent studies have shown that BIK1 interacts with receptors for pathogen- or damage-associated molecular patterns such as FLS2 and PEPRs to regulate immune response against different types of pathogens [69–73] and defense response to insect pests [74]. The *bik1* mutant plants showed an altered root architecture [68], similar to morphological phenotypes often seen in mutants with Pi starvation response [75], indicating a possible involvement of *BIK1* in Pi starvation response in Arabidopsis. Therefore, we investigated whether BIK1 functions in Pi starvation response and our results demonstrate that BIK1 plays a role in regulation of Pi homeostasis in Arabidopsis.

## Results

### Responsiveness of *BIK1* to Pi starvation

**Fig. 1:**
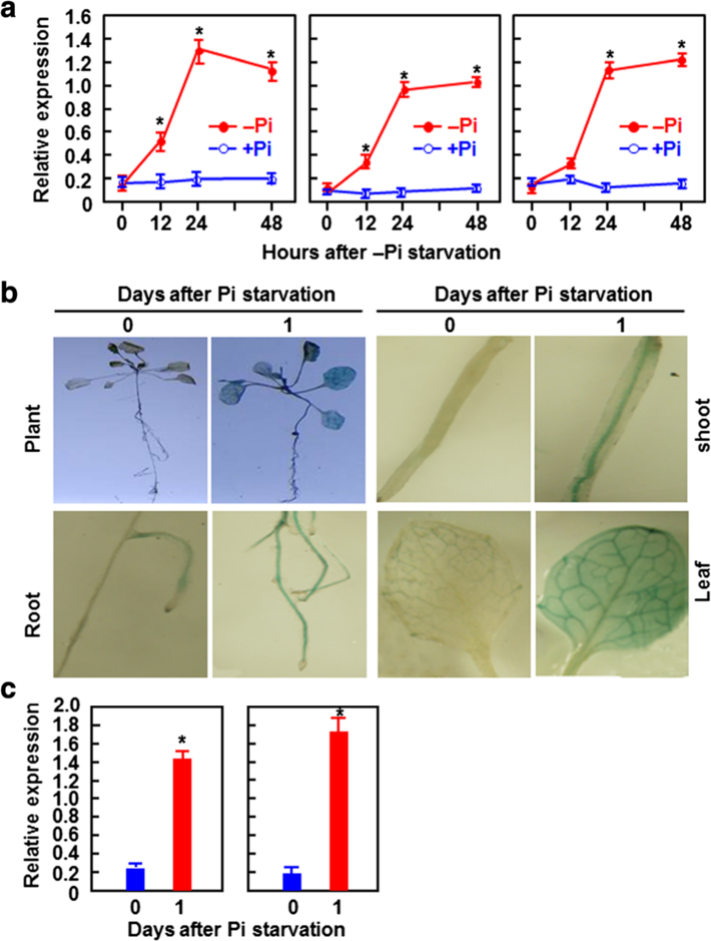
Responsiveness of *BIK1* to Pi starvation. **a** Expression changes of *BIK1* in different tissues of WT plants under + Pi and –Pi conditions. Seven-day-old seedlings grown hydroponically under normal Pi condition were transferred to medium supplemented with Pi (+Pi, 250 μM) or without Pi (−Pi). Roots, shoots and leaves were collected for analysis of *BIK1* expression by qRT-PCR at indicated time points after transferring. **b** Detection of *BIK1* promoter activity in *BIK1*_*pro*_::GUS seedlings by GUS staining. **c** Expression changes of *GUS* gene in root and lead tissues of *BIK1*_*pro*_::GUS seedlings under –Pi condition. Seven-day-old *BIK1*_*pro*_::GUS seedlings grown in normal medium were transferred to medium without Pi supplement and samples were collected at 0 and 1 day after transferring for GUS staining and analysis of gene expression. Data were normalized with the transcript level of *UBQ10* and relative expression levels were shown as folds of the *UBQ10* level. Data presented are the means ± SD from three independent experiments and * above the error bars indicate significant differences at *p* < 0.05 level between the + Pi and –Pi conditions

When grown on MS medium under normal conditions, the *bik1* plants produced shorter primary roots and longer and significantly more root hairs and lateral roots than WT plants [68], which is reminiscent of the mutants with defects in Pi nutrition [75]. These observations led us to examine whether BIK1 has a function in Pi starvation response. We first examined whether *BIK1* is responsive to Pi starvation stress by analyzing the expression patterns of *BIK1* in seedlings grown under + Pi and –Pi conditions. As shown in Fig. 1a, expression of *BIK1* was detected in roots, shoots and leaves of seedlings grown under + Pi condition and no significant changes in *BIK1* expression was observed during the experiment period. However, expression level of *BIK1* was markedly induced with similar patterns in roots, shoots and leaves of seedlings after transferring to medium without Pi supplement (Fig. 1a). The transcript levels of *BIK1* in seedlings grown under –Pi condition increased at 12 h and peaked at 24 and 48 h after transferring, leading to 7.5 ~ 11.2 folds of increases over those in seedlings grown under + Pi condition (Fig. 1a). To gain further information on spatial expression of the *BIK1* gene in response to Pi starvation, we generated *BIK1*_*pro*_::GUS transgenic lines and compared the GUS staining patterns in T2 seedlings during Pi starvation stress. Slight GUS staining was observed in leaves and roots of the *BIK1*_*pro*_::GUS seedlings at 0 h after transferred to medium without Pi supplement (Fig. 1b), indicating a basal expression of *BIK1* in leaf and root tissues, similar to the results from RT-PCR. At 1 day after transferred to medium without Pi supplement, significant GUS staining was easily seen in roots, shoot and leaves of the *BIK1*_*pro*_::GUS seedlings (Fig. 1b). qRT-PCR analysis showed 7.3 and 9.7 folds of increases in the expression levels of *GUS* gene in root and leaf tissues of the *BIK1*_*pro*_::GUS seedlings at 1 day after transferring to –Pi condition (Fig. 1c). Notably, GUS staining was clearly observed in vascular tissues of roots, shoots and leaves of the *BIK1*_*pro*_::GUS seedlings subjected to Pi starvation (Fig. 1b). Together, data from RT-PCR and GUS staining demonstrate that *BIK1* is responsive to Pi starvation.

### Increased Pi concentration in *bik1* plants

**Fig. 2:**
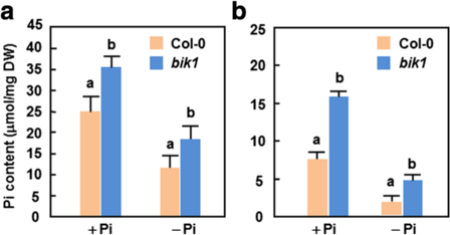
Increased total Pi content in *bik1* plants. Total Pi contents in roots (**a**) and leaves (**b**) of WT and *bik1* plants grown under + Pi (250 μM) and –Pi condition for 27 days were estimated. Data presented are the means ± SD from three independent experiments and different letters above the columns indicate significant differences at *p* < 0.05 level between same tissues of WT and *bik1* plants grown under same condition

We next examined whether loss of *BIK1* function affects Pi homeostasis in *bik1* plants. Total Pi contents in leaves and roots of WT and *bik1* plants grown hydroponically under + Pi (250 μM) and –Pi conditions for 27 days were measured. As shown in Fig. 2, a significant increase in total Pi contents was observed in leaves and roots of *bik1* plants as compared to WT plants under both + Pi and –Pi conditions. When grown under + Pi condition, total Pi contents in roots and leaves of *bik1* plants were 0.43 and 1.12 times higher over those in WT plants, respectively (Fig. 2a and b). Similarly, when grown under –Pi condition, total Pi contents in roots and leaves of *bik1* plants were 0.55 and 1.05 times higher than those of WT plants, respectively (Fig. 2a and b). These data indicate that BIK1 has a function either in Pi uptake or in the transfer of Pi from the roots to the leaves.

### Altered root architecture in *bik1* plants

**Fig. 3:**
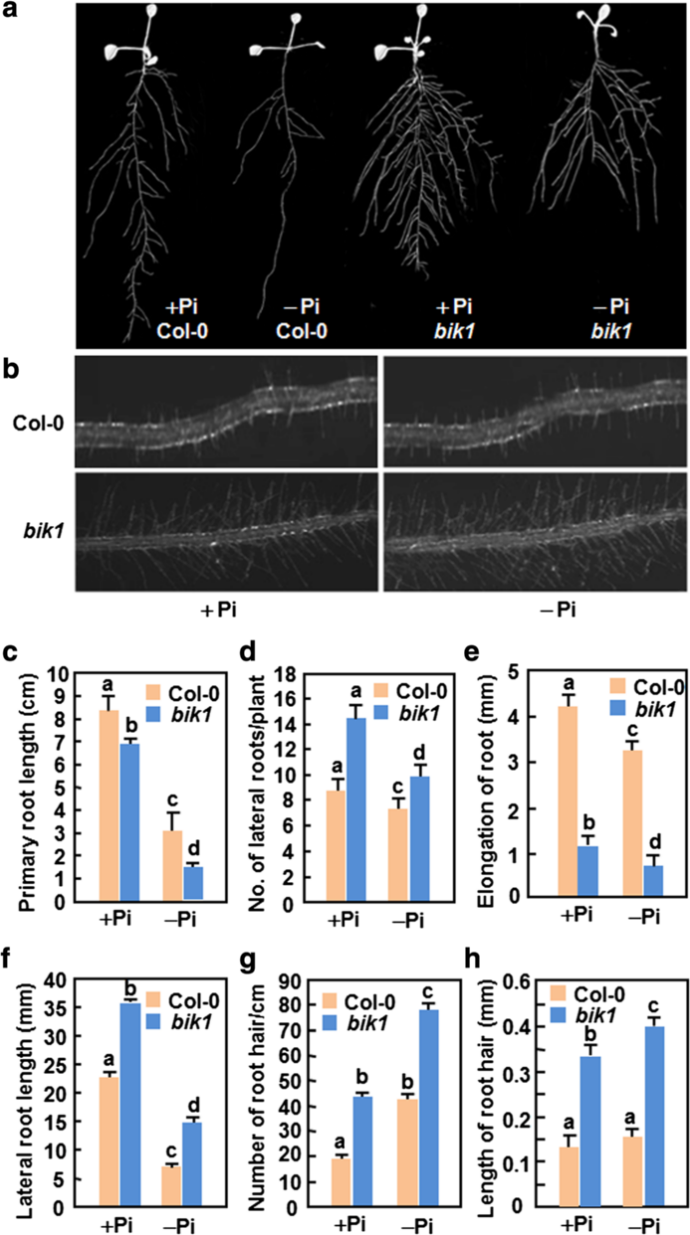
Altered root architecture in *bik1* seedlings grown under + Pi and –Pi conditions. Seven-day-old seedlings grown under normal condition were transferred into fresh medium supplemented with (+Pi, 250 μM) or without Pi (−Pi) and grown for another 7 days. **a** Root architecture in WT and *bik1* seedlings. **b** Root hairs in WT and *bik1* seedlings. Fragments of 5 mm from root tips were presented. **c** to **h** Comparative parameters for length of primary roots (**c**), numbers of lateral roots (**d**), elongation of primary roots (**e**) and lateral roots (**f**), and numbers (**g**) and length (**h**) of root hairs of WT and *bik1* seedlings grown under + Pi and –Pi condition. Data presented are the means ± SD from three independent experiments and different letters above the columns indicate significant differences at *p* < 0.05 level

Root architecture plays important roles in maintaining Pi homeostasisn in plants [75]. We compared the root architecture of WT and *bik1* seedlings grown under Pi normal (+Pi) and starvation (−Pi) conditions. When compared with WT seedlings, the *bik1* seedlings grown under + Pi condition showed shorter primary root and more lateral roots (Fig. 3a, c and d), and this trend was much evident in the *bik1* seedlings grown under –Pi condition (Fig. 3a, c and d). When grown under + Pi and –Pi conditions, the elongation rate of the primary and lateral roots of the *bik1* seedlings was markedly reduced as compared with WT seedlings (Fig. 3e and f). Furthermore, the *bik1* seedlings grown under + Pi and –Pi conditions showed more and longer root hairs than WT seedlings (Fig. 3b, g and h). These data indicate that BIK1 plays an important role in regulating development of root architecture.

### Increased anthocyanin accumulation and acid phosphatase activity in *bik1* plants

**Fig. 4:**
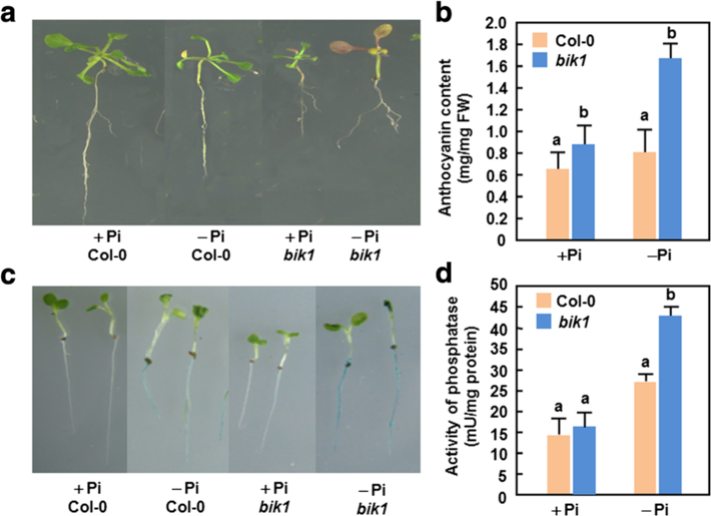
Increased levels of anthocyanin accumulation and acid phosphatases activity in *bik1* plants. **a** and **b** Measurements of anthocyanin contents. Seven-day-old WT and *bik1* seedlings grown on 1/2 MS were transferred into MS medium supplemented with Pi (+Pi, 1 mM) or without Pi (−Pi). Photos were taken and anthocyanin contents were measured at 5 days after treatment. **c** and **d** Detection of acid phosphatase activity. Ten-day-old seedlings grown in liquid medium were transferred to fresh medium supplemented with Pi (+Pi, 1 mM) or without Pi (−Pi) and covered with a layer of 0.008 % BCIP-containing agarose. Photos were taken and the activity of acid phosphatase in roots was estimated at 5 days after treatment. Data presented are the means ± SD from three independent experiments and different letters above the columns indicate significant differences at *p* < 0.05 level between WT and *bik1* plants grwon under same condition

Accumulation of anthocyanin and increased secretion of acid phosphatases are characteristic symptoms in plants under Pi starvation conditions [3]. We thus examined and compared the accumulation of anthocyanin and activity of acid phosphatase between WT and *bik1* plants grown under + Pi and –Pi conditions. When grown on medium under –Pi condition for 5 days, the leaves of the *bik1* seedlings turned to be purple while WT seedlings still kept green (Fig. 4a). The anthocyanin contents in *bik1* seedlings grown under + Pi and –Pi conditions were much higher than those in WT seedlings, showing 32 % and 105 % increase, respectively (Fig. 4b). No visible staining and significant changes in acid phosphatase activity were detected in WT and *bik1* seedlings grown + Pi condition (Fig. 4c and d). When grown under –Pi condition, roots of the *bik1* seedlings are stained deep blue while the staining of roots of WT seedlings is much lighter (Fig. 4c). Similarly, activity of acid phosphatase in roots of *bik1* seedlings is much higher than that in WT seedlings, grown under –Pi condition, showing an increase of ~65 % (Fig. 4d). These results indicate that the *bik1* seedlings secrete higher levels of acid phosphatase than WT seedlings under Pi starvation stress. Collectively, these results suggest that loss of *BIK1* function results in typical Pi starvation responses as revealed by the increased anthocyanin contents and acid phosphatase activity.

### Reduced ROS accumulation in *bik1* plants under Pi starvation condition

**Fig. 5:**
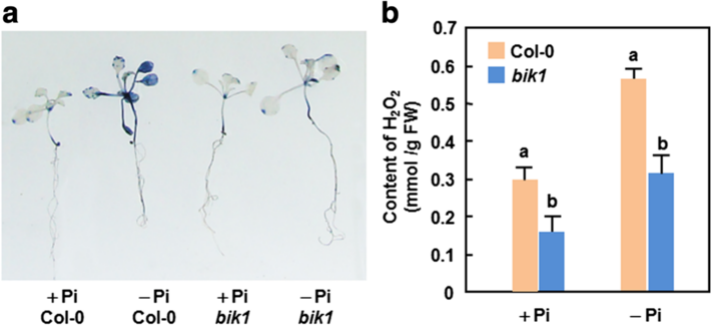
Reduced accumulation of ROS in *bik1* plants. Four-week-old seedlings grown under + Pi (250 μM) and –Pi conditions were collected for detection of superoxide anion and measurement of H_2_O_2_. **a** NBT staining of superoxide anion in WT and *bik1* plants grown under + Pi and –Pi conditions. **b** Quantification of H_2_O_2_ contents in WT and *bik1* plants grown under + Pi and –Pi conditions. Data presented are the means ± SD from three independent experiments and different letters above the columns indicate significant differences at *p* < 0.05 level between WT and *bik1* plants grwon under same condition

ROS, generated and accumulated in plant response to different types of stresses, has been implicated in many biological processes including biotic and abiotic responses [76, 77]. Therefore, we examined whether mutation in *BIK1* gene affects the balance of ROS and hence leads to accumulation of ROS in plants during Pi starvation stress. In NBT staining of superoxide anion, no significant difference was detected between WT and *bik1* plants grown under + Pi condition; however, significant accumulation of superoxide anion in leaves, stem and roots of WT and *bik1* plants grown under –Pi condition was observed (Fig. 5a). Notably, accumulation of superoxide anion in WT plants was much higher than that in *bik1* plants grown under –Pi condition (Fig. 5a). Quantification of H_2_O_2_ contents revealed a significant lower level of H_2_O_2_ accumulated in *bik1* plants than that in WT plants when grown under + Pi condition (Fig. 5b). Accumulation of H_2_O_2_ in WT and *bik1* plants grown under –Pi condition was markedly increased, leading to 95 and 78 % of increases as compared with those in plants grown under + Pi condition, respectively (Fig. 5b). Under –Pi condition, the content of H_2_O_2_ in *bik1* plants was approximately 54 % of that in WT plants (Fig. 5b). These results indicate that loss of *BIK1* function led to reduced accumulation of ROS in *bik1* plants under –Pi starvation stress.

### Altered expression of Pi starvation-induced genes and miRNA399 in *bik1* plants

**Fig. 6:**
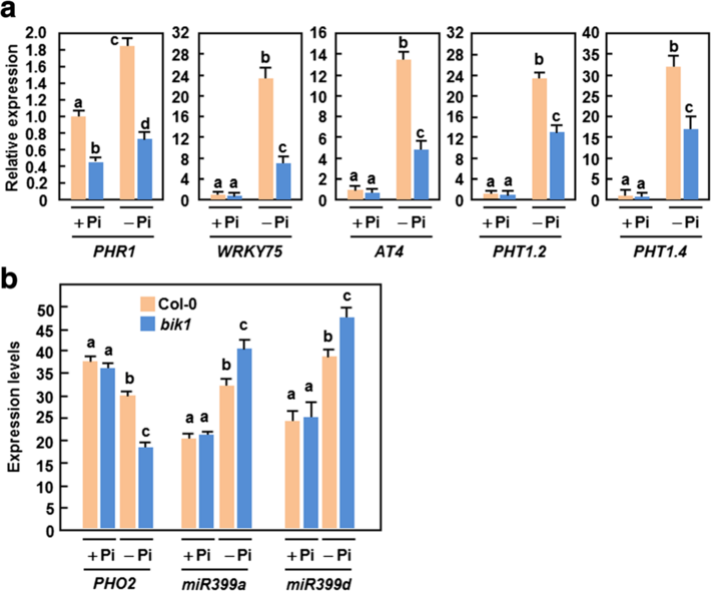
Down-regulated expression of Pi starvation-induced genes in *bik1* plants. **a** Expression patterns of Pi starvation-induced genes in WT and *bik1* seedlings grown under + Pi and –Pi conditions. Seven-day-old seedlings grown under normal Pi condition were treated with Pi (+Pi, 250 μM) or without Pi (−Pi) for 5 days and total RNA were extracted from root tissues at 5 days after treatment. **b** Expression patterns of *PHO2* and *miRNA399* in WT and *bik1* seedlings grown under + Pi and –Pi conditions. Seven-day-old seedlings grown under normal Pi condition were transferred to medium supplemented with Pi (+Pi, 250 μM) or without Pi (−Pi) and samples were collected at 48 h after treatment. Expression of Pi starvation-responsive genes and miR399s was analyzed by qRT-PCR using specific primers and data were normalized with the transcript level of *UBQ10* as an internal control. Data presented are the means ± SD from three independent experiments and different letters above the columns indicate significant differences at *p* < 0.05 level

Expression of some well-characterized Pi starvation-induced genes was analyzed and compared between WT and *bik1* seedlings that had been transferred into medium with Pi (+Pi) or without Pi (−Pi) for 5 days. When grown under + Pi condition, the expression levels of *AT4* [78], *WRKY75* [39], *PHT1;2* and *PHT1;4* [24, 28] in *bik1* seedlings were comparable to those in WT seedlings, while the expression of *PHR1* [37] in *bik1* seedlings was reduced (Fig. 6a). The expression levels of the Pi starvation-induced genes tested were markedly increased in WT and *bik1* plants grown under –Pi condition (Fig. 6a). However, significant reductions in the expression levels of these Pi starvation-induced genes was observed in *bik1* plants, as compared with those in WT plants, under –Pi condition (Fig. 6a). These results indicate that mutation in *BIK1* leads to reduced expression of the Pi starvation-induced genes in plants grown under –Pi condition.

It was found that expression of *PHO2*, encoding a ubiquitin-conjugating E2 enzyme, was suppressed by *miRNA399*, which controls Pi homeostasis in plants and whose expression is up-regulated by Pi starvation [48–52]. To examine whether *PHO2*/*miRNA399* links to increased accumulation of Pi in *bik1* plants, we analyzed the changes in levels of *PHO2* expression and two miRNA399 primary transcripts, *miRNA399a* and *miRNA399d*, in *bik1* seedlings grown under + Pi and –Pi conditions. The expression level of *PHO2* and the transcript levels of *miRNA399a* and *miRNA399d* were comparable in WT and *bik1* seedlings grown under + Pi condition. However, the expression levels of *PHO* in WT and *bik1* seedlings under –Pi condition were significantly reduced whereas the transcript levels of *miRNA399a* and *miRNA399d* in WT and *bik1* seedlings grown under –Pi condition were markedly increased, as compared with the corresponding seedlings under + Pi condition (Fig. 6b). A further reduction in the expression of *PHO2* and a further increase in the transcripts of *miRNA399a* and *miRNA399d* in *bik1* seedlings was observed as compared with those in WT seedlings grown under –Pi condition (Fig. 6b). Together, these data indicate that the *PHO2*/*miRNA399* plays a role in regulating Pi accumulation in *bik1* plants under –Pi condition.

## Discussion

The maintenance of cellular Pi homeostasis in plants involves complicated regulatory mechanisms. Several studies have demonstrated that posttranslational modifications such as phosphorylation, sumoylation and ubiquitination of regulatory proteins play critical roles in Pi starvation responses [54, 55, 57]. Recently, genome-wide co-expression analysis identifies dozens of protein kinases as important regulators of Pi deficiency-induced root hair remodeling [79, 80]. It was also found that *in vivo* phosphorylation activation of PPC1 (phosphoenolpyruvate carboxylase 1) is involved in the metabolic adaptations of Pi starvation [81] and activation of MKK9-MPK3/MPK6 enhances phosphate acquisition in Arabidopsis [56]. These findings indicate an important role for protein kinases in regulating Pi starvation response. In this study, we found that BIK1, a plasma membrane-localized receptor-like protein kinase [68], plays an important role in modulating Pi starvation responses and functions as a negative regulator of Pi homeostasis in Arabidopsis plants. Thus, evidence presented in the current study renders BIK1 for a novel function in Pi starvation response, in addition to its previously reported functions in immunity [68–73].

Several lines of evidence presented in this study support that BIK1 functions in Pi starvation response. Earlier study has shown that expression of *BIK1* could be induced by infection with *B. cinerea* as well as treatments with some well-known defense signaling molecules [68]. Our qRT-PCR analysis of *BIK1* expression and determination of *BIK1* promoter activity in stable *BIK1*_*pro*_::GUS transgenic seedlings demonstrate that *BIK1* can be induced by Pi starvation and Pi starvation-induced expression of *BIK1* in root and shoot tissues initiated earlier than in leaf tissues (Fig. 1a). The pattern of *BIK1* expression induced by Pi starvation is similar to most of the Pi starvation-induced genes identified so far, like *WKRY75*, *ERF070* and *ZAT6* [39, 40, 44]. Importantly, promoter activity analysis revealed that GUS staining driven by the *BIK1* promoter was initiated in vascular tissues of root (Fig. 1b) that is the primary organ that responds to Pi starvation, further confirming the responsiveness of *BIK1* to Pi starvation. Thus, it is likely that *BIK1* can respond rapidly to the altered Pi status of plants under Pi starvation condition. It was particularly noteworthy that BIK1 is a plasma membrane-localized receptor-like protein kinase and has been shown to be phosphorylated by other kinases (e.g. BAK1 and FLS2) for its full activity in plant immune responses [69, 70]. In this context, it is thus possible that BIK1 is phosphorylated by unknown kinase during Pi starvation stress and phosphorylated BIK1 acts in Pi starvation response, in addition to its transcriptional regulation upon Pi starvation.

The primary root, lateral root, and root hairs are three main components of the root architecture that is critical to absorption of Pi from soil. Low P availability can drastically alter the root architecture by switching the indeterminate growth to determinate growth to promote lateral root growth [75]. Generally, not only the primary roots but also almost all mature lateral roots enter into the determinate developmental program under low Pi condition [82]. The *bik1* seedlings showed a significant change in root architecture and the primary root length of *bik1* plants was significantly decreased (Fig. 6). This is similar to a common phenomenon observed that the primary root length is decreased significantly due to the Pi starvation-induced determinate growth in primary root [82]. In addition to the change of the primary root, the *bik1* plants showed increased number and length of lateral roots and root hairs (Fig. 6), which increase root surface contacting an increased soil volume to explore Pi availability in soil [75]. Similar root architectures were observed in the *bik1* plants under + Pi and –Pi conditions (Fig. 6). Thus, it is likely that the effect of BIK1 on the development of root architecture is independent of the Pi status in plants and Pi availability in soil. Nevertheless, the characteristic root architecture observed in *bik1* plants suggests that BIK1 is a negative regulator of lateral root and root hair development. This is in agreement with previous observations that the *bik1* plants showed some defects in growth and development, e.g. weak stem strength, early flowering and less seed setting [68]. Together, these data further demonstrate that BIK1 is required for normal plant growth and development [68]. It will be interesting to investigate whether the growth and developmental defects in *bik1* plants is due to an activated Pi starvation response and if this is the case, the results obtained will further support a cross-talk between Pi starvation response and plant growth/development. It was previously found that overexpression of *ZAT6* retards growth and results in typical Pi starvation responses [40].

Accompanied with high contents of total Pi in roots and leaves of *bik1* plants under + Pi and –Pi conditions (Fig. 2) are the significant accumulation of anthocyanin (Fig. 4a and b), increased activity of acid phosphatase (Fig. 4a and b) and alterations in expression of Pi starvation-induced genes (Fig. 6). Collectively, these data imply that, whatever grown under + Pi condition or under –Pi condition, a Pi starvation response including those of physiological, molecular and metabolic changes is activated in *bik1* plants. Increased activity of acid phosphatases in *bik1* plants grown under –Pi condition (Fig. 3b) not only represents a characteristic Pi starvation response [83] but also can release more Pi available for absorption by the roots. This is supported by the findings that mutations in genes encoding for purple acid phosphatases affect markedly the uptake of Pi from exogenous sources [84–86]. Because expression of *BIK1* was rapidly induced by Pi starvation (Fig. 1), it seems possible that BIK1 is involved in Pi uptake by regulating the activity of acid phosphatase. Similar observations were also obtained for some Pi starvation response regulators such as PHR1 and ZAT6, which have been shown to affect activity of acid phosphatase and Pi uptake in transgenic plants with altered expression of these genes [40, 85, 87, 88]. Interestingly, expression of *Pht1;2* and *Pht1;4*, encoding for high-affinity Pi transporters [24, 28], *WRKY75*, encoding a WRKY transcriptional factor involved in Pi acquisition [39], *At4*, a member of the Mt4/TPSI1 gene family involved in Pi distribution [78], and *PHR1*, encoding a MYB transcriptional factor involved in Pi starvation response signaling [37], was suppressed significantly in roots of *bik1* plants under –Pi condition (Fig. 6a). These data demonstrate that BIK1 has global effect on a set of Pi starvation-induced genes that are involved in Pi acquisition and mobilization. Notably, the expression levels of the tested Pi starvation-responsive gene except *PHR1* in *bik1* plants were slightly reduced as compared to those in WT plants under + Pi condition (Fig. 6a), indicating a possibility that these Pi starvation-responsive genes may be also affected by mutation of *BIK1* itself. Particularly, the expression of *PHR1* in *bik1* plants grown under + Pi condition was significantly reduced as compared to that in WT plants (Fig. 6a), leading to an open question whether BIK1 acts upstream of PHR1 in regulating the Pi starvation signaling. This can be clarified by phenotyping and analysis of *PHR1* expression in *BIK1*-overexpressing plants and/or detailed examination of biochemical and genetic requirement of BIK1 for Pi starvation response. It is currently difficult to link the function of BIK1 to Pi uptake or its root-to-leaf translocation as Pi uptake and transportation in WT plants is also affected under –Pi condition (Fig. 2). This can further be explained by the reduced expression of these tested Pi starvation-induced genes in root of *bik1* plants as compared to those in WT plants under –Pi condition (Fig. 6). Furthermore, altered expression patterns of *PHO2* and *miRNA399a/d*, which are thought to be involved in systemic signaling of Pi starvation response and Pi distribution in plants [49, 52], were also observed in *bik1* plants grown under + Pi and –Pi conditions (Fig. 6b). Thus, it is likely that BIK1 is involved in maintaining Pi homeostasis in whole plants. Taken together, these data support an idea that BIK1 is a negative regulator of Pi starvation responses, probably through affecting development of root architecture and a series of physiological and biochemical events related to Pi acquisition, mobilization and translocation. It is reasonable to speculate that, under Pi starvation stress, the *bik1* plants may experience a reduced Pi content as the WT plants but they can uptake efficiently Pi from growth environment with their significantly increased root surface area, leading to an increased Pi content in root and leaf tissues of *bik1* plants (Fig. 2). However, the detailed mechanism that BIK1 functions in Pi starvation response remains to be explored further. Particularly, characterization of the targets that are phosphorylated by BIK1 will be greatly helpful in elucidating the early signaling events that determine Pi starvation response.

ROS not only are toxic compounds produced in plant response to various stresses but also plays an integral role as signaling molecules in regulation of numerous biological processes such as growth, development, and responses to biotic and/or abiotic stress [89–91]. The involvement of ROS in Pi starvation response has been established recently. In this study, we found that both WT and *bik1* plants grown under –Pi condition accumulated more ROS, represented by H_2_O_2_ and superoxide anion, than those in plants grown under + Pi condition (Fig. 5). This is similar to the previous observations that H_2_O_2_ concentrations in roots increased upon Pi deprivation [92]. Notably, the accumulation of superoxide anion in WT and *bik1* plants under + Pi condition was comparable, the accumulation of superoxide anion in *bik1* plants was significantly reduced as compared to that in WT plants under –Pi condition (Fig. 5). By contrast, the *bik1* plants accumulated lower level of H_2_O_2_ than WT plants grown under + Pi condition (Fig. 5), which is similar to the histochemical staining in soil-grown *bik*1 plants [68]. Thus, it is likely that BIK1 affects the accumulation of superoxide anion and H_2_O_2_ in different ways: loss of *BIK1* function suppressed, at least partially, the Pi starvation-induced accumulation of superoxide anion while BIK1 regulates directly the accumulation of H_2_O_2_ in plants under normal condition. This may also imply different functions of superoxide anion and H_2_O_2_ in Pi starvation response. On the other hand, increased accumulation of ROS in plants under –Pi condition might be one of the stress responses as those in response to other abiotic stress [90]. It was recently shown that localization pattern of ROS accumulated in root during Pi starvation stress is critical to shape the root architecture [93–96]. The reduced accumulation of ROS in *bik1* plants in relative to those in WT plants (Fig. 5), which is similar to the observation that loss of *BIK1* function impaired the PAMP-induced ROS burst in immunity [97, 98], may attribute to the altered root architecture of *bik1* plants (Fig. 3). However, detailed analysis of ROS localization and the possible mechanism regulating ROS generation in root of *bik1* plants will provide new insights into the connection between BIK1 and ROS in development of root architecture.

## Conclusions

In summary, this study demonstrates that BIK1 is a Pi starvation-responsive gene and functions as a negative regulator of Pi homeostasis in Arabidopsis. This not only renders a novel function for BIK1 but also strengthens our understanding of post-transcriptional regulation during Pi starvation responses in plants. Considering that BIK1 is a plasma membrane-localized receptor-like protein kinase, it thus may play a role in sensing and/or processing of the Pi starvation signal during early stage of Pi starvation stress.

## Methods

### Plant materials and growth condition

*Arabidopsis thaliana* ecotype Columbia (Col-0) and *bik1* (provided by Dr. Tesfaye Mengiste, Purdue University, USA) mutant [68] were used in this study. Seeds were surface sterilized with 70 % ethanol, washed with sterilized distill water and vernalized at 4 °C for 2 days before germination. Hydroponic, solid medium and liquid medium cultivation were used for different purpose of experiments. For solid medium cultivation, the basic medium used contained 2.06 mM NH_4_NO_3_, 1.88 mM KNO_3_, 0.31 mM MgSO_4_, 0.1 mM MnSO_4_, 0.03 mM ZnSO_4_, 0.1 mM CuSO_4_, 0.3 mM CaCl_2_, 5.0 mM KI, 0.1 mM CoCl_2_, 0.1 mM FeSO_4_; 0.1 mM EDTA, 0.1 mM H_3_BO_3_, 1 mM Na_2_MoO_4_.2H_2_O, 3 g/L sucrose, 10 g/L agar, pH 5.8. When treated for Pi sufficiency (+Pi) and Pi deficiency (−Pi), the medium was supplemented with 1 mM KH_2_PO_4_, 10 μM KH_2_PO_4_ and 0.99 mM KCl. For hydroponic cultivation, seedlings at 5 ~ 7-leaf stage were transferred to 1/2 Hoagland solution for short adaption and then transferred to hydroponic solution containing 250 μM Pi or no Pi [99]. For liquid medium cultivation, seeds were dispensed in 1/2 MS medium without agar and rinsed with sterilized distilled water at 7 days. Seedlings were then transferred in MS liquid medium with Pi (1 mM) or without Pi and allowed to grow with shaking at 85 rpm. Plants were grown in a growth room under a 16 h light (100 μmol · s^−1^ · m^−2^ photons m^−2^ sec^−1^ of intensity) and 8 h dark cycle at 22 ± 2 °C with 60 % relative humidity.

### Measurement of root system architecture

Seven-day-old seedlings grown on 1/2 MS medium were transferred to Petri dishes and treated for + Pi or –Pi for another 7 days. Length of main root, number and length of the lateral root were measured. Total numbers of root hairs in a 5 mm region from root tip were recorded. Data were recorded from 15 individual plants from each treatment.

### Anthocyanin analysis

Measurement of anthocyanin was performed as described previously [100]. Briefly, seedlings grown under + Pi or –Pi condition in solid medium were put into 20 mL extraction solution (propanol : HCl : H_2_O = 18:1:81, V/V/V) and boiled for 90 s. The mixtures were kept overnight in dark at room temperature, centrifuged for 40 min at 5000× g, and the absorbance (A) was measured at 535 nm and 650 nm. The A_535_ values were corrected with the A_650_ values using formula A_535_ = A_535_-2.2 × A_650_. Anthocyanin contents were calculated according to a standard curve prepared with the same protocol.

### Quantification of total Pi content

Total Pi content was quantified according to the U.S. Environmental Protection Agency Method 365.2 with minor modifications [39]. Briefly, samples (~50 mg/sample) were flamed to ash after recording of their dry weights and then 100 μL of concentrated HCl was added. Ten microliters of the mixture were drawn and diluted into 790 μL of water, followed by addition of 200 μL assay solution (4.8 mM NH_4_MoO_4_, 2.5 N H_2_SO_4_, and 35 μM ascorbic acid). The reactions were incubated at 45 °C for 20 min and the absorbance at 650 nm was measured spectrophotometrically. Contents of total Pi were calculated according to the Pi standard curve prepared with the same procedure.

### Quantification and staining of acid phosphatase activity

Activity of acid phosphatase was quantified using the pNPP hydrolysis assay according to a previously described method [101]. Briefly, 30 mg of samples were grounded, transferred to Eppendorf tubes and then spin for 10 min at 2000 × g. Reactions containing 100 μL supernatant, 100 μL *p*-nitrophenol sodium phosphate and 2.8 mL buffer were kept at 30 °C for 10 min, with shaking occasionally, and terminated by addition of 1 mL 0.5 M NaOH. The absorbance of the reactions was determined spectrophotometrically at 400 nm and the activity of acid phosphatase was calculated from the production of *p*-nitrophenol. Total protein was estimated using Bradford’s reagent and the total acid phosphatase activity was expressed as mU/mg protein. Staining of acid phosphatase activity was performed as described by Tomscha et al. [102] with minor modifications. Ten-day-old seedlings grown in liquid medium were rinsed in –Pi medium, transferred to fresh medium supplemented with Pi (1 mM) or without Pi and then covered with a layer of 0.05 % agarose solution containing 0.008 % 5-bromo-4-chloro-3-indolyl phosphate (BCIP) [103].

### Detection and quantification of H_2_O_2_

For quantification of H_2_O_2_, 30 mg samples from 4-week-old plants were completely ground, followed by addition of 200 μL 20 mM K_2_HPO_4_ (pH6.5) phosphate buffer. Quantification of H_2_O_2_ was performed using a commericial kit (Jiancheng Bioengineering Institute, Nanjing, China) according to the manufactruer’s recommendation. Detection of superoxide anion was performed by the nitroblue tetrazolium (NBT) staining [104]. Four-week-old seedlings were vacuum infiltrated with 2 ml of 10 mM potassium phosphate buffer (pH 7.5) containing 10 mM NaN_3_ and 0.1 % NBT for 30 min, cleared by boiling in 96 % ethanol, remained in 50 % ethanol before taking photos.

### qRT-PCR analysis of gene expression

Total RNA was extracted using TRIZOL reagent (Invitrogen, Shanghai, China) according to the manufacturer’s instructions. First strand cDNA was synthesized from 500 ng of total RNA using SuperScript III Kit (Invitrogen, Shanghai, China). The qPCR reactions contained 12.5 μL SYBR Premix Ex TaqTM (TaKaRa, Dalian, China), 0.1 μg cDNA and 7.5 pmol of each of gene-specific primers in 25 μL and were conducted on a CFX96 real-time PCR system (BioRad, Hercules, CA, USA). Gene-specific primers used were as followings: BIK1-q-F, 5′-ACT TAT GGG TAC GCC GCG CCT GAG T-3′; BIK1-q-R, 5′-GGC ACG GAC CAC TTG GTC CA-3′; GUS-q-1F, 5′-AGG TGC ACG GGA ATG TTT CG-3′; GUS-q-1R, 5′-TGT GAG AGT CGC AGA ACA TT-3′; PHR1-q-1F, 5′-GTG ATT GGC ATG AAT GGG CTG AC-3′; PHR1-q-1R, 5′-CGC AAT TCC ACA GAC GGA GAA GG-3′; AT4-q-1F, 5′-GAT CGA AGT TGC CCA AAC GA -3′; AT4-q-1R, 5′-GAG CGA TGA AGA TTG CAT GAA G-3′; WRKY75-q-1F, 5′-GAG AAA TCC ACC GAA AAC TTC GAG CAT AT-3′; WRKY75-q-1R, 5′-GCA TGG TTT TTC TTT TCA ACA CAC GTA AAA TGT A-3′; PHT1.2-q-1F, 5′-AGG GCA AGT CCC TCG AAG AAC T-3′; PHT1.2-q-1R, 5′-ATC AAA CAA ACC ACA AAC AAC TCC ACA T-3′; PHT1.4-q-1F, 5′-TTG CTC CTA ATT TTC CTG ATG CT -3′; PHT1.4-q-1R, 5′-TGT GCC GGC CGA AAT CT-3′. Pri-miRNA399a-1F, 5′-TGG CAG GAA ACC ATT ACT TAG ATC T-3′; Pri-miRNA399a-1R, 5′-TCA CTA ATT AAA AGC AAT GCA TAA AGA GA-3′; Pri-miRNA399d-1F, 5′-TTA CTG GGC GAA TAC TCC TAT GG-3′; Pri-miRNA399d-1R, 5′-ATT TTA CTT GCA TAT CTA GCC AAT GC-3′; PHO2-q-1F, 5′-AGG TTT GAA GCT CCA CCC TCA-3′; PHO2-q-1R, 5′-CCC AAG ATG TGA TTG GAG TTC C-3′; UBQ10-q-1F, 5′-GGC CTT GTA TAA TCC CTG ATG AAT AAG-3′; UBQ10-q-1R, 5′-AAA GAG ATA ACA GGA ACG GAA ACA TAG T-3′. Relative gene expression levels were calculated using 2^–△△CT^ method with three independent biological replicates.

### Generation of *BIK1*_*pro*_::GUS transgenic line and GUS staining

A 2 kb sequence upstream of the *BIK1* start codon was PCR amplified using primers AtBIK1-GUS-1F (5′-ATA **CTG CAG** CTT GTT GAT TGA TTA ATA GAT TAC C-3, a *Pst*I site in bold) and AtBIK1-GUS-1R (5′-GCC **GGA TCC **AGA ACT GAA GCA AGA ACC CAT C-3′, a *Bam*HI site in bold) and cloned into vector pCAMBIA1301. Transformation of wild-type Col-0 plants was performed using the floral dip infiltration method mediated by *Agrobacterium tumefaciens* strain GV3101. Plants of T2 generations from kanamycin-resistant transformants were used for GUS histochemical staining [105].

## Abbreviations

+Pi, Pi sufficiency; *B. cinerea*, *Botrytis cinerea*; BCIP, 5-bromo-4-chloro-3-indolyl phosphate; *BIK1*, *Botrytis-induced kinase1*; MKK, mitogen-activated protein kinase kinase; MPK, mitogen-activated protein kinase; NBT, nitroblue tetrazolium; PAMP, pathogen associated molecular pattern; *Pht1*, *PHOSPHATE TRANSPORTER1*; Pi, phosphate; −Pi, Pi deficiency; pNPP, *p*-nitrophenol sodium phosphate; *PPC1*, *phosphoenolpyruvate carboxylase 1*; qRT-PCR, quantitative reverse transcription PCR; ROS, reactive oxygen species; WT, wild type
